# Acinetobacter baumannii Targets Human Carcinoembryonic Antigen-Related Cell Adhesion Molecules (CEACAMs) for Invasion of Pneumocytes

**DOI:** 10.1128/mSystems.00604-20

**Published:** 2020-12-22

**Authors:** Cecilia Ambrosi, Daniela Scribano, Meysam Sarshar, Carlo Zagaglia, Bernhard B. Singer, Anna Teresa Palamara

**Affiliations:** a IRCCS San Raffaele Pisana, Department of Human Sciences and Promotion of the Quality of Life, San Raffaele Roma Open University, Rome, Italy; b Department of Public Health and Infectious Diseases, Sapienza University of Rome, Rome, Italy; c Dani Di Giò Foundation-Onlus, Rome, Italy; d Department of Public Health and Infectious Diseases, Sapienza University of Rome, Laboratory affiliated to Institute Pasteur Italia-Cenci Bolognetti Foundation, Rome, Italy; e Microbiology Research Center (MRC), Pasteur Institute of Iran, Tehran, Iran; f Research Laboratories, Bambino Gesù Children’s Hospital, IRCCS, Rome, Italy; g Institute of Anatomy, Medical Faculty, University Duisburg-Essen, Essen, Germany; University of California, San Francisco

**Keywords:** *Acinetobacter baumannii*, carcinoembryonic antigen‐related cell adhesion molecules, bacterial adhesion/invasion, MAPKs, Rubicon

## Abstract

Multidrug-resistant Acinetobacter baumannii is regarded as a life-threatening pathogen mainly associated with nosocomial and community-acquired pneumonia. Here, we show that A. baumannii can bind the human carcinoembryonic antigen-related cell adhesion molecule (CEACAM) receptors CEACAM1, CEACAM5, and CEACAM6. This specific interaction enhances A. baumannii internalization in membrane-bound vacuoles, promptly decorated with Rab5, Rab7, and lipidated microtubule-associated protein light chain 3 (LC3). Dissecting intracellular signaling pathways revealed that infected pneumocytes trigger interleukin-8 (IL-8) secretion via the extracellular signal-regulated kinase (ERK)1/2 and nuclear factor-kappa B (NF-κB) signaling pathways for A. baumannii clearance. However, in CEACAM1-L-expressing cells, IL-8 secretion lasts only 24 h, possibly due to an A. baumannii-dependent effect on the CEACAM1-L intracellular domain. Conversely, the glycosylphosphatidylinositol-anchored CEACAM5 and CEACAM6 activate the c-Jun NH_2_-terminal kinase (JNK)1/2-Rubicon-NOX2 pathway, suggestive of LC3-associated phagocytosis. Overall, our data show for the first time novel mechanisms of adhesion to and invasion of pneumocytes by A. baumannii via CEACAM-dependent signaling pathways that eventually lead to bacterial killing. These findings suggest that CEACAM upregulation could put patients at increased risk of lower respiratory tract infection by A. baumannii.

**IMPORTANCE** This work shows for the first time that Acinetobacter baumannii binds to carcinoembryonic antigen-related cell adhesion molecule 1 (CEACAM1), CEACAM5, and CEACAM6. This binding significantly enhances A. baumannii internalization within alveolar host cell epithelia. Intracellular trafficking involves typical Rab5 and Rab7 vacuolar proteins as well as light chain 3 (LC3) and slowly progresses to bacterial killing by endosome acidification. CEACAM engagement by A. baumannii leads to distinct and specific downstream signaling pathways. The CEACAM1 pathway finely tunes interleukin-8 (IL-8) secretion, whereas CEACAM5 and CEACAM6 mediate LC3-associated phagocytosis. The present study provides new insights into A. baumannii-host interactions and could represent a promising therapeutic strategy to reduce pulmonary infections caused by this pathogen.

## Abstract (precis)

This work shows for the first time that Acinetobacter baumannii binds to carcinoembryonic antigen-related cell adhesion molecule 1 (CEACAM1), CEACAM5, and CEACAM6. This binding significantly enhances A. baumannii internalization within alveolar host cell epithelia.

## INTRODUCTION

Acinetobacter baumannii is a Gram-negative bacterium that has emerged in recent decades as an important opportunistic pathogen worldwide (1). Hospitals and communities become the favorite ecological niches of A. baumannii, where it is responsible for a broad range of nosocomial infections, predominantly ventilator-associated and community-acquired pneumonia as well as bloodstream infections (2, 3). A. baumannii infections involving multidrug-resistant (MDR) strains occur mainly in intensive care unit (ICU) patients suffering from an underlying disease or immune suppression or who have undergone major surgical procedures (3–7). Several studies highlighted that A. baumannii is able to express several virulence factors, including those required for biofilm formation, desiccation resistance, secretion systems, micronutrient acquisition systems, and twitching motility, as well as adherence to and invasion of human epithelial cells (1, 8–11). The first essential step to successfully establish an infection is bacterial adhesion to host cells. This fundamental process is mediated by adhesins distinguished in nonfimbrial and fimbrial adhesins (12). The latter adhesins are located at the tip of the fimbriae and are often referred to as lectins since they recognize oligosaccharide residues of glycoprotein or glycolipid receptors on host cells (12). Conversely, monomeric or trimeric nonfimbrial adhesins are surface-exposed proteins that bind mainly to extracellular matrix (ECM) proteins (12, 13). Surface adhesins that play a role in the adhesion to human epithelial cells include the giant cell surface biofilm-associated protein (Bap), the Acinetobacter trimeric autotransporter (Ata), the filamentous hemagglutinin adhesin FhaB, the trimeric outer membrane porin OmpA, and porinD (14–18). The latter phosphorylcholine-containing protein was shown to be involved in A. baumannii adhesion to and internalization into membrane-bound vacuoles within epithelial cells via the platelet-activating factor receptor (PAFR) (17). Outer membrane proteins (Omps) are known to play important roles in bacterial pathogenicity (19). So far, several Omps responsible for the binding of carcinoembryonic antigen (CEA)-related cell adhesion molecules (CEACAMs) were found in a number of bacterial pathogens (20, 21). CEACAMs belong to a group of immunoglobulin (Ig)-related glycoproteins and are involved in several cellular processes, such as cell adhesion, intracellular and intercellular signaling, inflammation, and cancer progression (22). These molecules are associated with the membrane through either a hydrophobic transmembrane domain or a glycosylphosphatidylinositol (GPI) lipid moiety, while the surface-exposed N terminus is composed by a variable Ig-like domain followed by 0 to 6 constant Ig-like domains (22, 23). Several family members, including CEACAM1, CEACAM5, and CEACAM6, are widely distributed on epithelial tissue surfaces throughout the human body (22–24). CEACAM1, CEACAM5, and CEACAM6 can be coexpressed and individually expressed (24). Various bacterial human pathogens were shown to engage CEACAMs for tight adhesion to epithelial cells, thereby allowing bacterial colonization and multiplication (20, 25). CEACAM-binding pathogens include Escherichia coli, Neisseria meningitidis, Neisseria gonorrhoeae, Haemophilus influenzae, Moraxella catarrhalis, *Salmonella* spp., Helicobacter pylori, and *Fusobacterium* spp. (20, 25). Furthermore, CEACAM engagement triggers bacterial uptake, dictates the intracellular trafficking of pathogens in host cells, and inhibits proinflammatory action (20, 21, 25–31). Since A. baumannii mechanisms of adhesion to and internalization into host cells are largely uncharacterized, the present study was undertaken to assess CEACAM involvement in adhesion to and invasion of pneumocytes and their contribution in A. baumannii intracellular trafficking. Here, we demonstrate for the first time that CEACAM1, CEACAM5, and CEACAM6 provide enhanced host cell adhesion to A. baumannii and trigger its uptake into epithelial cells. Moreover, we provide the first evidence of two distinct and specific CEACAM-dependent intracellular signaling pathways that, eventually, lead to bacterial killing.

## RESULTS

### A. baumannii cells bind CEACAM receptors to enhance host cell invasion.

**FIG 1:**
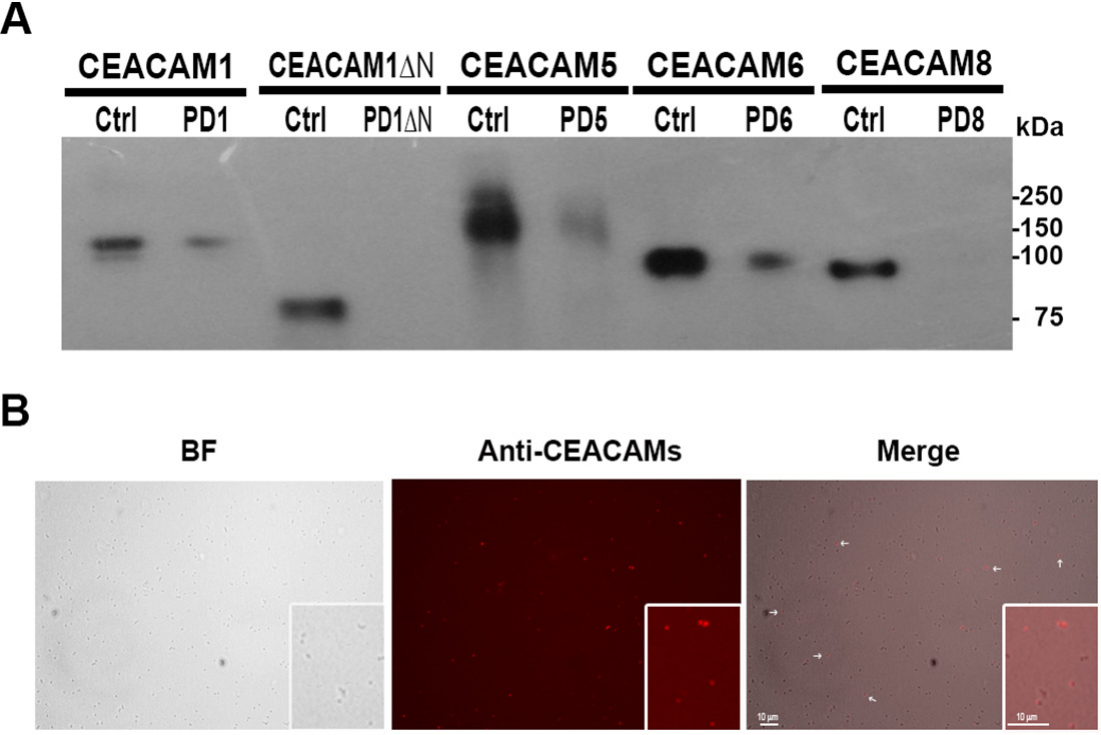
A. baumannii binds to CEACAM receptors. Strain AB5075 grown to exponential phase was incubated with 10 µg of the indicated recombinant proteins and subjected to pulldown. (A) Pulldown samples (PD) were resolved by Tricine-PAGE and visualized by Western blotting using the anti-CEACAM antibodies (MAb 6G5j and C5-1X) and HRP-conjugated secondary antibodies. Purified proteins (50 ng) were loaded as controls (Ctrl). The recombinant CEACAM1 protein lacking the complete N-domain (CEACAM1ΔN) was also assayed. One representative experiment of four is shown. (B) Pulldown bacteria incubated with MAb 6G5j and TRITC-labeled secondary antibodies were centrifuged on polylysine-treated coverslips and mounted for bright-field (BF) and fluorescence microscopic analyses. One representative experiment of four is shown.

To investigate if CEACAM molecules are targeted by A. baumannii cells, purified recombinant human CEACAMs were used in pulldown experiments with A. baumannii strain AB5075. Interestingly, a positive interaction with CEACAM1-Fc, CEACAM5-Fc, and CEACAM6-Fc, but not with CEACAM8-Fc, was detected by Western blotting after pulldown (Fig. 1A). Since the majority of the bacterial-CEACAM1 interactions are mediated by its N-terminal IgV-like domain, a pulldown experiment using a recombinant human CEACAM1-Fc protein lacking this domain (CEACAM1ΔN) was performed. As shown in Fig. 1A, no interaction could be observed, indicating that the lack of the N-domain abrogates A. baumannii-CEACAM1 interaction. The binding of CEACAM1-Fc, CEACAM5-Fc, and CEACAM6-Fc and A. baumannii cells was also confirmed by fluorescence immunostaining with an anti-CEACAM antibody following incubation of bacteria with purified recombinant CEACAMs (Fig. 1B). No immunofluorescent signal could be detected in bacteria incubated with CEACAM8-Fc or untreated bacteria (data not shown).

**FIG 2:**
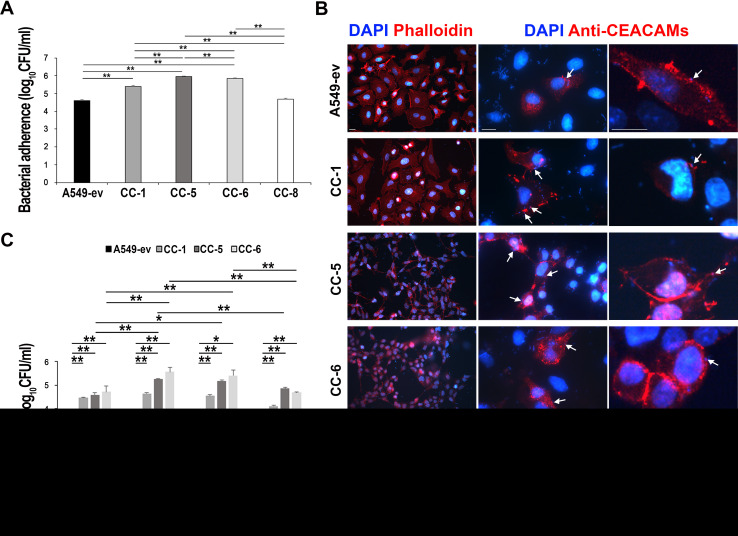
Expression of CEACAM receptors increases A. baumannii adherence to and invasion of lung epithelial cells. Stably transfected A549 cell lines expressing CEACAM1 (CC-1), CEACAM5 (CC-5), CEACAM6 (CC-6), and CEACAM8 (CC-8) as well as the control cell line transfected with the empty vector (A549-ev) were infected with strain AB5075 at an MOI of 1 for the adhesion time (2.5 h). (A) Following extensive washing, the number of adherent bacteria was assessed by counting the CFU/ml. Each adherence assay was performed in duplicate wells, and all data are given as the mean ± standard deviation (SD) from five independent experiments. Asterisks represent *P* values evaluated by one-way analysis of variance (ANOVA); **, *P* < 0.01. (B) Representative images of parallel cell monolayers stained in red with phalloidin or immune-labeled with the anti-CEACAM antibody (MAb 6G5j), respectively, followed by DAPI staining (blue). Arrows indicate examples of CEACAMs colocalized with bacteria. Two different magnifications are presented. Images were acquired with the digital FireWire color camera DFX300. Scale bars = 10 µm. (C) A549, CC-1, CC-5, and CC-6 were infected as described above. Following the adhesion time (2.5 h), colistin sulfate (10 µg/ml) was added to kill extracellular bacteria. After a further 24, 48, 72, and 96 h of incubation, the number of intracellular bacteria was assessed by counting the CFU/ml. Each invasion assay was performed in duplicate wells, and all data are given as the mean ± standard deviation (SD) from five independent experiments. Asterisks represent *P* values evaluated by one-way ANOVA; *, *P* < 0.05; **, *P* < 0.01.

The human lung adenocarcinoma cell line A549 represents a well-established model for studying A. baumannii infections (14, 17, 32, 33). To evaluate the adhesion rates of A. baumannii to CEACAM receptors, stably transfected A549 cells individually expressing CEACAM1-L, CEACAM5, CEACAM6, and CEACAM8 (referred to as CC-1, CC-5, CC-6, and CC-8, respectively) as well as the control cell line, A549 transfected with the empty vector (A549-ev), were used in the *in vitro* adherence assay (34; this study). It is important to note that, as the cell lines were kept in the proliferating stage, they did not express their endogenous CEACAM1, CEACAM5, and CEACAM6 (34). No significant differences in the levels of CEACAMs expressed by transfected cells were observed (Fig. S1). Cell monolayers were infected with strain AB5075 (multiplicity of infection [MOI] 1) for 2.5 h, and cell-associated bacteria were evaluated quantitatively by CFU (CFU/ml) and qualitatively by fluorescence staining and indirect immunofluorescence using specific antibodies (Fig. 2). Remarkably, A. baumannii strain AB5075 adhered strongly to CC-5- and CC-6-expressing cells and, to a lesser extent, to CC-1; in contrast, bacterial adherence to CC-8 and control cells was very scarce (Fig. 2A). Moreover, immunofluorescence experiments showed that A. baumannii cells colocalized with CEACAM receptors, corroborating their interaction (Fig. 2B). Altogether, these results indicate for the first time a direct interaction between A. baumannii strain AB5075 cells and CEACAM1, CEACAM5, and CEACAM6 receptors.

10.1128/mSystems.00604-20.1 FIG S1 Surface expression of CEACAMs by flow cytometry in the A549 cell line. The A549 cell line was stably transfected with CEACAM1, CEACAM5, CEACAM6, and CEACAM8, as described in Materials and Methods. The A549 cell line carrying the empty vector was used as the control throughout the study. The surface expression of each CEACAM was determined by flow cytometry in individual clones growing in log phase using the MAb 6G5j antibody (FACS calibur; BD). Download FIG S1, TIF file, 0.2 MB. Copyright © 2020 Ambrosi et al. 2020 Ambrosi et al. This content is distributed under the terms of the Creative Commons Attribution 4.0 International license.

To evaluate the involvement of CEACAMs in A. baumannii internalization, an antibiotic protection assay using colistin was performed. The stably transfected CEACAM cell lines were infected with strain AB5075 (MOI 1) for 24, 48, 72, and 96 h, and the number of internalized viable bacteria was assessed (CFU/ml) at each time point (Fig. 2C). No bacterial growth was detected on LB agar plates supplemented with colistin, ruling out the onset of resistant bacteria. Statistically significant differences in the efficiency of bacterial internalization were evident between the control cell line and those expressing individual CEACAM receptors (Fig. 2A and C). Therefore, these results strengthen the involvement of these receptors in mediating bacterial adhesion and internalization. Interestingly, bacterial intracellular replication rates showed that the number of intracellular bacteria increased during the first 48 h and remained unchanged after 72 h of infection, starting to drop significantly at 96 h postinfection (Fig. 2C). No morphological changes, such as shrinkage, cytoplasmic vacuolization, plasma membrane blebbing, and chromatin condensation, were observed either in infected cells at 96 h postinfection or in uninfected cells. These data prompted us to investigate A. baumannii intracellular trafficking and CEACAM-dependent cellular signaling in response to invading bacteria.

### Rab5, Rab7, and LC3 decorate A. baumannii membrane-bound vacuoles at early times postinfection.

**FIG 3:**
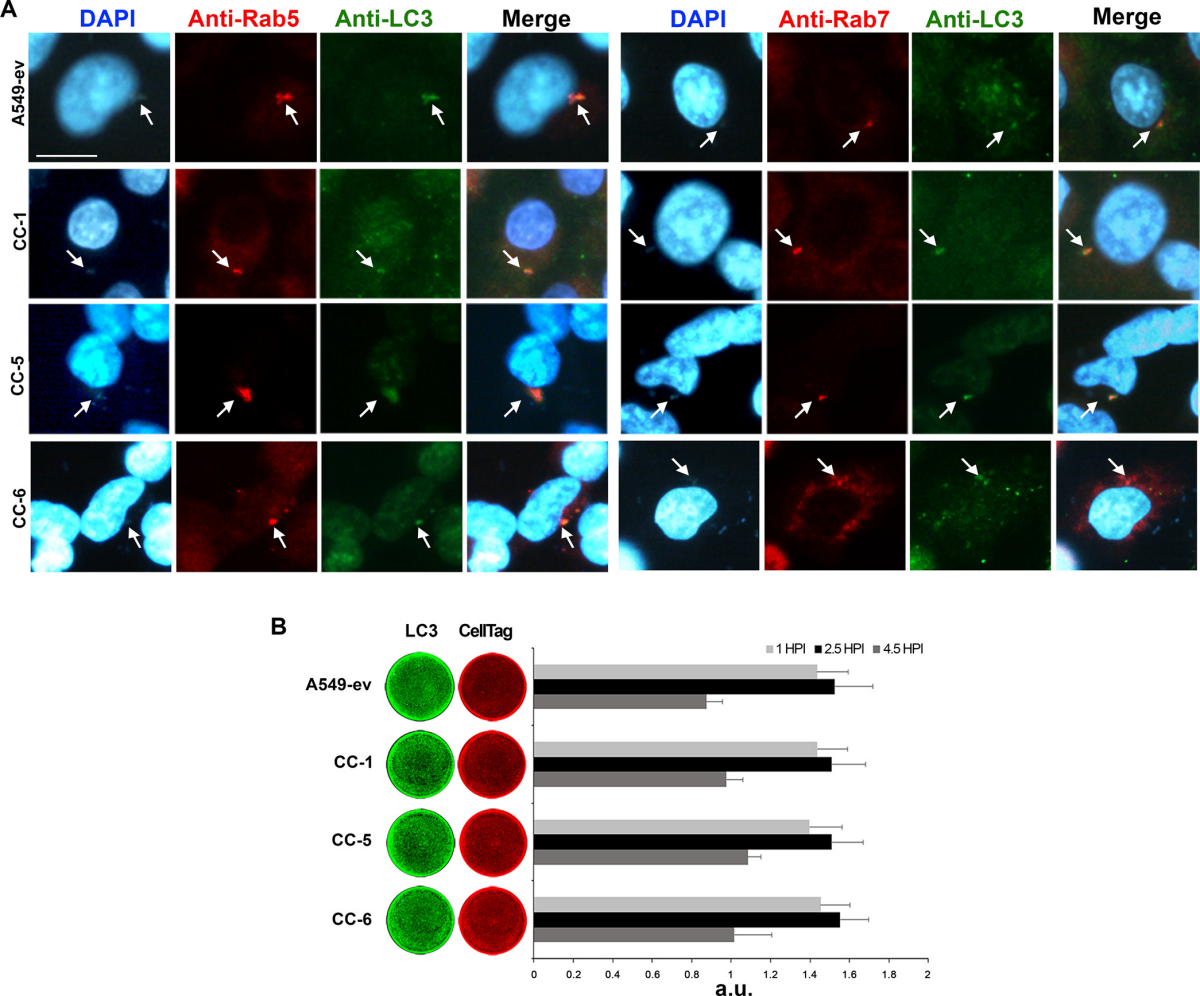
A. baumannii colocalizes with endocytic markers and LC3. Stably transfected A549 cell lines expressing CEACAM1 (CC-1), CEACAM5 (CC-5), and CEACAM6 (CC-6) as well as the control cell line transfected with the empty vector (A549-ev) were infected with strain AB5075 at an MOI of 1 for the adhesion time (2.5 h), followed by a further 2 h of incubation in the presence of colistin sulfate (10 µg/ml) to kill extracellular bacteria. (A) Cell monolayers were fixed and processed for immunofluorescence labeling with anti-Rab5, -Rab7, and -LC3 antibodies and were DAPI stained. Representative images were acquired with the digital FireWire color camera DFX300. Scale bar = 10 µm. (B) Parallel cell monolayers infected as described above were fixed after 1 and 2.5 h and a further 2 h of incubation in the presence of colistin sulfate prior to being hybridized with anti-LC3 antibody (green). CellTag 700 staining (red) was used to quantify cell monolayers. Plates were scanned using an Odyssey infrared imager, and signals of the integrated fluorescence intensity were acquired using LI-COR Image Studio 3.1 software. HPI, hours postinfection. Results are expressed as arbitrary units (a.u.) and represent means ± standard deviation (SD) of four independent experiments done in triplicate.

Previous studies demonstrated that A. baumannii cells are internalized and survived within membrane-bound vacuoles in the cytoplasm (17, 35). Following phagocytosis of microorganisms, Rab G-proteins are fundamental coordinators of intracellular trafficking vesicles (36). Among this family of proteins, the small GTPase Rab5 regulates clathrin-coated vesicle formation and the early events of the phagocytic pathway, while Rab7 is specifically related to degradative compartments (36). To understand the early events of A. baumannii infection, A549 cell monolayers expressing individual CEACAMs were infected with strain AB5075 (MOI 1) for 2.5 h and, after 2 h of colistin protection assay, were fixed and immunolabelled using the antibodies anti-Rab5 and -Rab7 (Fig. 3A). A. baumannii localized with Rab5- and Rab7-labeled compartments in all cell lines, but the extent of colocalization in A549-ev was very low due to the little number of internalized bacteria (Fig. 3A). As Rab5 localizes within early endocytic compartments and Rab7 is sequentially recruited at later times, we concluded that A. baumannii transits via the normal endocytic/phagocytic pathway after cell invasion. Since it was previously reported that the A. baumannii OmpA protein induces the expression of lipidated microtubule-associated protein light chain 3 (LC3) (37), infected A549-CEACAM cell lines were immunostained with an anti-LC3 antibody (Fig. 3A). Indirect immunofluorescence results showed that LC3 colocalized with both Rab5 and Rab7 in all cell lines (Fig. 3A). The same pattern was observed during a similar experiment using an mRFP-GFP/LC3 expressing cell line, in which the chimeric LC3-GFP is overexpressed; however, in this cell line, as for the A549-ev, the percentage of internalized bacteria was dramatically low (Fig. S2). These results indicate that LC3 decorates the A. baumannii membrane-bound vesicles early on after internalization and during the Rab5‐to‐Rab7 switch. Next, a time course quantification analysis of total LC3 was performed (Fig. 3B). Results show that LC3 started to accumulate and increased at 1 h and 2.5 h postinfection, respectively, and then decreased after 4.5 h in all infected cell lines (Fig. 3B). Overall, these results indicate that early on after internalization, A. baumannii interacts with the endocytic pathway and the LC3 protein.

10.1128/mSystems.00604-20.2 FIG S2 A. baumannii colocalizes with endocytic markers and LC3 in HeLa cells. mRFP-GFP-LC3 HeLa cells (HeLa/LC3-GFP) were infected with strain AB5075 at an MOI of 1 for 2.5 h (adhesion time), followed by a further 2-h incubation in the presence of colistin sulfate (10 µg/ml) to kill extracellular bacteria. Cell monolayers were fixed and processed for immunofluorescence labeling with anti-Rab5 and were DAPI stained. Representative images of mRFP-GFP-LC3 HeLa cells were acquired with the digital FireWire color camera DFX300. Scale bar = 10 µm. Download FIG S2, TIF file, 1.2 MB. Copyright © 2020 Ambrosi et al. 2020 Ambrosi et al. This content is distributed under the terms of the Creative Commons Attribution 4.0 International license.

### Intracellular A. baumannii cells survive the harsh environment of membrane-bound vacuoles.

**FIG 4:**
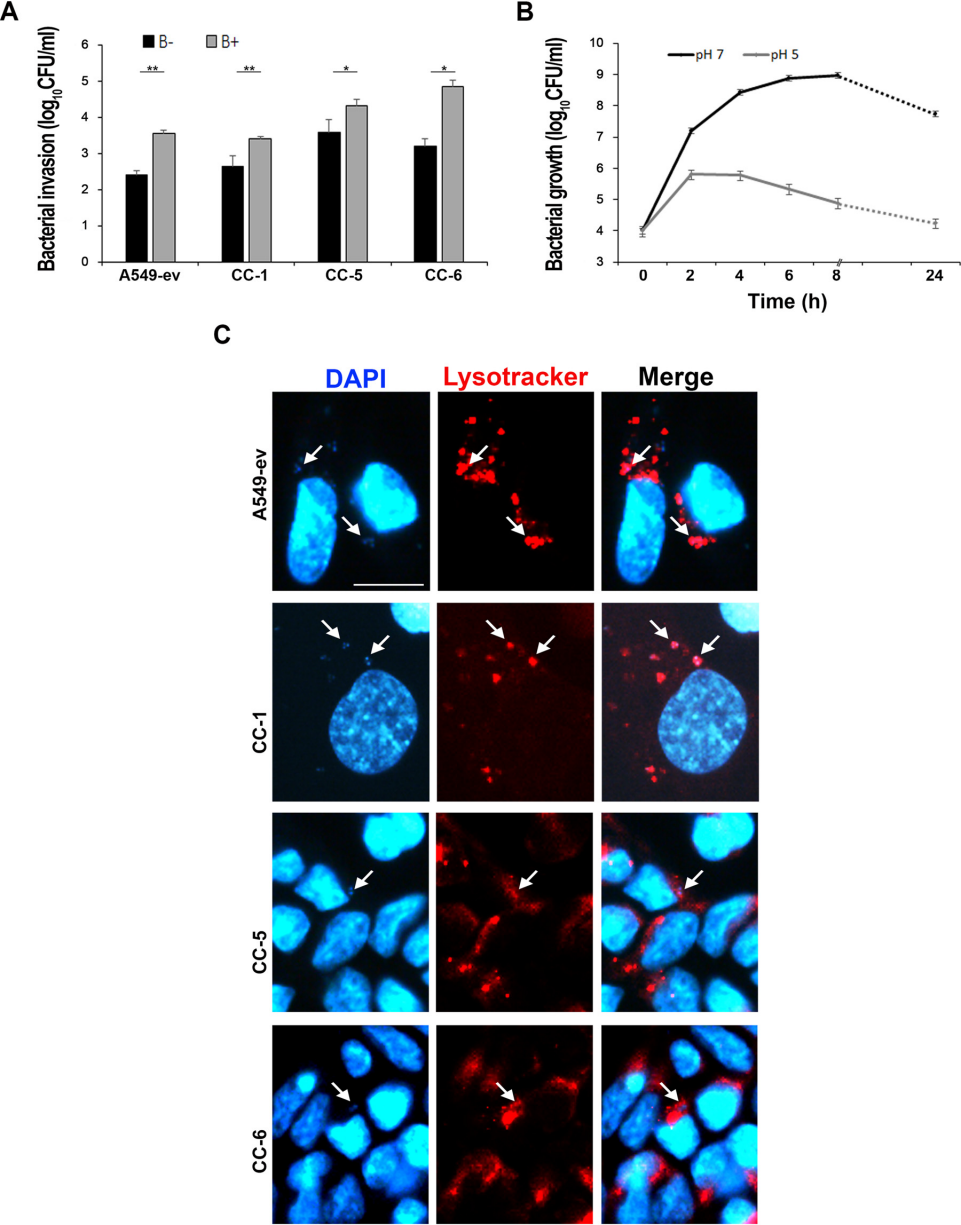
Acidification has detrimental effects on A. baumannii viability. (A) Stably transfected A549 cell lines expressing CEACAM1 (CC-1), CEACAM5 (CC-5), and CEACAM6 (CC-6) as well as the control cell line transfected with the empty vector (A549-ev) were treated with the V-ATPase inhibitor bafilomycin A1 (100 nM) (B+) or left untreated (B–) prior to being infected with strain AB5075 at an MOI of 1. Following the adhesion time (2.5 h) and a further 2 h in the presence of colistin sulfate to kill extracellular bacteria, infected cell monolayers were lysed, and the number of intracellular bacteria was assessed by counting the CFU/ml. Each invasion assay was performed in duplicate wells, and all data are given as the mean ± SD from four independent experiments. Asterisks represent *P* values evaluated with Student’s *t* test, *, *P* < 0.05; **, *P* < 0.01. (B) Strain AB5075 was grown in LB medium under neutral and acidic pH conditions. At the indicated time points, the number of viable bacteria was assessed by counting the CFU/ml. (C) Stably transfected A549 cell lines, CC-1, CC-5, and CC-6 as well as the control cell line A549-ev were infected with strain AB5075 at an MOI of 1. Following the adhesion time (2.5 h), colistin sulfate (10 µg/ml) was added. After a further 72 h of incubation, Lysotracker red was used to stain acidic vesicles. Cell monolayers were fixed and DAPI stained. Representative images were acquired with the digital FireWire color camera DFX300. Scale bar = 10 µm.

Upon Rab5 recruitment, the endocytic membrane starts to accumulate vacuolar (V)-ATPases that translocate protons within this compartment to decrease the pH to 6.1-6.5 (36). The gradual accumulation of active V‐ATPases over time allows further vesicle acidification, attaining a pH value of 5-5.5 (36). To assess the effect of vacuole acidification on A. baumannii viability, a specific V-ATPase inhibitor, bafilomycin A1 was used (38). A549 cell monolayers expressing individual CEACAMs were infected as reported above. Bafilomycin A1 was added at the time of infection and kept in the medium for the entire incubation period. Untreated uninfected and infected cells served as controls. No effect on morphology or viability in bafilomycin A1-treated cells was detected during or after the experiment (Fig. S3). Interestingly, bafilomycin A1 treatment increased the number of intracellular A. baumannii in comparison to untreated controls, thereby indicating that ATPase activity significantly affects A. baumannii intracellular multiplication (Fig. 4A). To determine A. baumannii growth rates under acidic conditions, strain AB5075 was grown in LB at pH 5 and 7, and the number of viable bacteria was assessed over time (Fig. 4B). Growth curves highlighted that A. baumannii multiplication rates were profoundly affected by the acidic medium, although bacteria showed a moderate degree of tolerance during the initial 4-h exposure (Fig. 4B). Acidification of the A. baumannii membrane-bound vacuoles was checked in infected A549 cell monolayers expressing individual CEACAMs by the use of LysoTracker red, a fluorescent acidotropic probe that labels any acidic compartment within cells. A. baumannii within endocytic vacuoles colocalized with LysoTracker at 72 and 96 h postinfection (Fig. 4C and data not shown). Overall, these results indicate that vacuoles enclosing A. baumannii undergo progressive acidification, and the steady number of intracellular bacteria at 72 h postinfection indicates that A. baumannii causes a delay of the detrimental vacuolar acidification, a key step of the host cell microbicidal response, for at least 48 h postinfection (Fig. 2C).

10.1128/mSystems.00604-20.3 FIG S3 Treatment with bafilomycin A1 does not affect cell morphology or viability. (A) Stably transfected A549 cell lines expressing CEACAM1 (CC-1), CEACAM5 (CC-5), and CEACAM6 (CC-6) as well as the control cell line transfected with the empty vector (A549-ev) were treated with bafilomycin A1 (100 nM) (B+) and infected with strain AB5075 at an MOI of 1 for 2.5 h (adhesion time), followed by a further 2-h incubation in the presence of colistin sulfate (10 µg/ml) to kill extracellular bacteria. Infected, bafilomycin A1-untreated cells (B−) were used as controls. Cell monolayers were fixed and DAPI/phalloidin stained to visualize cell morphology. Representative images were acquired with the digital FireWire color camera DFX300. Scale bar = 10 µm. (B) Cell viability was evaluated in bafilomycin A1-treated (B+) and untreated (B−) cells using a 3-(4,5-dimethylthiazol-2-yl)-2, 5-diphenyl tetrazolium bromide (MTT) assay (Sigma Aldrich, Rome, Italy). No statistical significance between treated and untreated cell monolayers was found (*P* value > 0.05). Download FIG S3, TIF file, 2.7 MB. Copyright © 2020 Ambrosi et al. 2020 Ambrosi et al. This content is distributed under the terms of the Creative Commons Attribution 4.0 International license.

### CEACAMs mediate distinct modulations of mitogen activated protein kinase (MAPK) signaling pathways.

**FIG 5:**
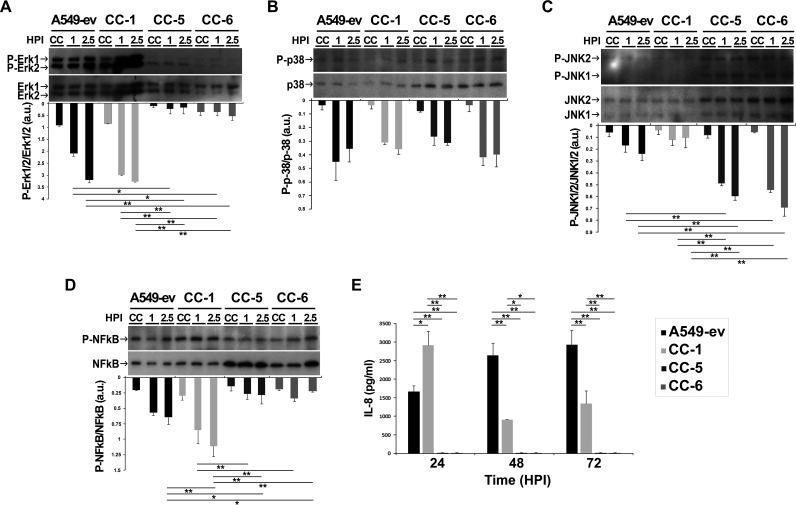
CEACAMs transduce different signaling pathways upon A. baumannii engagement. Stably transfected A549 cell lines expressing CEACAM1 (CC-1), CEACAM5 (CC-5), and CEACAM6 (CC-6) as well as the control cell line transfected with the empty vector (A549-ev) were infected with strain AB5075 at an MOI of 1 for the indicated times. (A to D) At each time point (hours postinfection [HPI]), whole-cell lysates in duplicate were prepared and used in Western blot analysis to detect (A) phospho (P)-ERK, ERK, (B) P-p38, p38, (C) P-JNK, JNK, and (D) P-NF-κB, NF-κB using specific antibodies. Images are representative, whereas bars depict the calculated ratios of phosphorylated/total protein content quantified by densitometry (ImageJ software) and expressed as arbitrary units (a.u.). (E) After 24, 48, and 72 h of infection, IL-8 production was measured in cell supernatants by sandwich enzyme-linked immunosorbent assay (ELISA). Data are given as the mean ± standard deviation (SD) from four independent experiments. Asterisks represent *P* values evaluated by one-way ANOVA, *, *P* < 0.05; **, *P* < 0.01.

CEACAMs are involved in several signal-transduction pathways, including those involving MAPKs (39). To investigate whether the engagement of CEACAM receptors by A. baumannii induces MAPK activation, a time course experiment was performed. A549 cell lines expressing individual CEACAMs were infected with strain AB5075 (MOI 1), and whole-cell extracts were analyzed by Western blotting at 1 h and 2.5 h postinfection using anti-phospho-extracellular signal-regulated kinase (ERK1/2), anti-phospho-p38, and anti-phospho-c-Jun NH_2_-terminal kinase (JNK1/2) antibodies (Fig. 5). Uninfected cells served as negative controls. Results show that adherent A. baumannii induced a strong and time-dependent increase in the phosphorylation of ERK1/2 in CC-1 and control cell lines (Fig. 5A). Conversely, ERK1/2 was not phosphorylated at any time during A. baumannii infection of CC-5 and CC-6 (Fig. 5A). No differences in the levels of phosphorylated p38 were detected among infected cell lines (Fig. 5B). On the other hand, a significant increase in the phosphorylation of JNK1/2 was observed only in infected CC-5 and CC-6 cell lines but not in CC-1 (Fig. 5C). Heat-killed bacteria induced a transient phosphorylation of MAPKs at 1 h postinfection, decreasing at the later time point (data not shown); this result indicates that only live bacteria were capable of sustaining durable MAPK activation. Since the three MAPKs might induce the expression of interleukin-8 (IL-8) via activation of nuclear factor-kappa B (NF-κB) (40), the levels of activated NF-κB and secreted IL-8 were assessed in whole-cell extracts by Western blotting and in supernatants by enzyme-linked immunosorbent assay (ELISA), respectively (Fig. 5D and E). Uninfected cells served as negative controls. Both the control and CC-1 cell lines showed an NF-κB activation pattern that paralleled the one seen for ERK1/2 (Fig. 5A and D). Interestingly, the CC-1 cell line secreted larger amounts of IL-8 than A549-ev control cells at 24 h postinfection, whereas they showed an opposite trend at later time points (Fig. 5E). Conversely, in both CC-5 and CC-6 cell lines, the levels of secreted IL-8 were comparable with those of uninfected cells (Fig. 5E).

**FIG 6:**
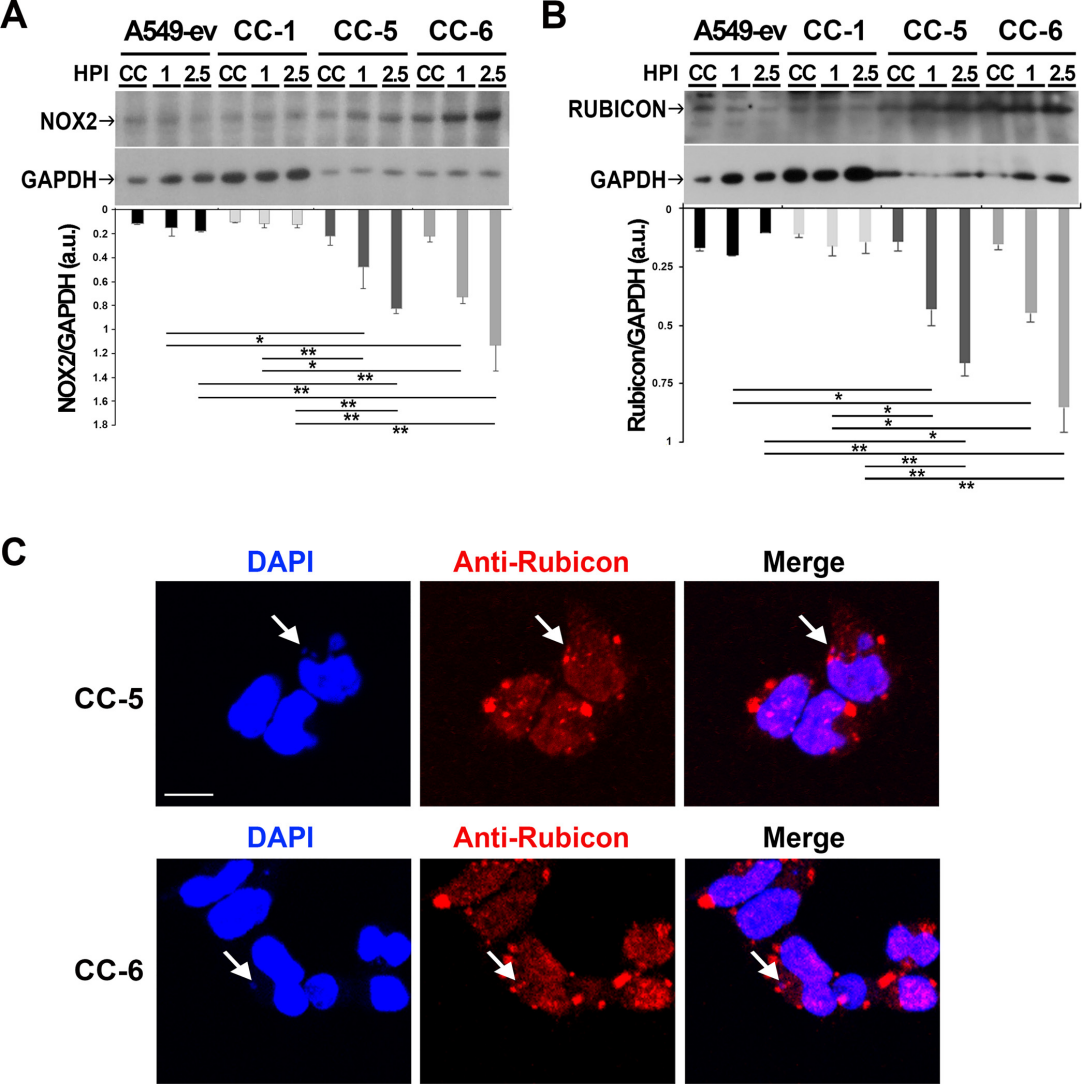
The CEACAM5 and CEACAM6 signaling pathway increases NOX2 and Rubicon expression upon A. baumannii engagement. Stably transfected A549 cell lines expressing CEACAM1 (CC-1), CEACAM5 (CC-5), and CEACAM6 (CC-6) as well as the control cell line transfected with the empty vector (A549-ev) were infected with strain AB5075 at an MOI of 1 for the indicated times. (A and B) At each time point (hours postinfection [HPI]), whole-cell lysates in duplicate were prepared and used in Western blot analysis to detect (A) NOX2 and (B) Rubicon using specific antibodies, while glyceraldehyde-3-phosphate dehydrogenase (GAPDH) was used as a loading control. Images are representative, whereas bars depict the calculated ratios of NOX2/GADPH and Rubicon/GAPDH quantified by densitometry (ImageJ software) and expressed as arbitrary units (a.u.). Data are given as the mean ± standard deviation (SD) from four independent experiments. Asterisks represent *P* values evaluated by one-way ANOVA; *, *P* < 0.05; **, *P* < 0.01. (C) CC-5 and CC-6 cell monolayers were infected with strain AB5075 at an MOI of 1 for the adhesion time (2.5 h), followed by a further 2 h of incubation in the presence of colistin sulfate (10 µg/ml) to kill extracellular bacteria. Cell monolayers were fixed and processed for immunofluorescence labeling with the anti-Rubicon antibody and were DAPI stained. Representative images were acquired with the DS-Qi2 monochrome camera. Scale bar = 10 µm.

The differences in the levels of activated JNK and the lack of IL-8 secretion in infected CC-5 and CC-6 cell lines led us to hypothesize that these receptors could induce a distinct signaling response via JNK. During the past years, a particular antimicrobial pathway has been discovered known as LC3-associated phagocytosis (LAP) (41). This autophagic-related pathway is triggered by surface receptors and requires Rubicon (RUN domain protein as Beclin-1 interacting and cysteine-rich containing) to promote endocytic trafficking and endosome maturation by controlling phosphatidylinositol 3-phosphate (PI3P) localization, active NOX2 complex stabilization, and Rab7 recruitment (41). Therefore, the expression levels of Rubicon and NOX2 were evaluated in the same whole-cell extracts by Western blotting (Fig. 6A and B). Interestingly, the expression levels of both Rubicon and NOX2 were enhanced at 1 h postinfection and showed further increases at 2.5 h postinfection in both CC-5 and CC-6 cell lines, while their expression was low in CC-1 and control cell lines (Fig. 6A and B). To investigate if bacteria localized with Rubicon at the same time point as Rab5 and Rab7, an indirect immunofluorescence was performed. Results showed that A. baumannii colocalized with Rubicon in both CC-5 and CC-6 (Fig. 6C), whereas no such colocalization was seen in CC-1 and control cells (data not shown).

Next, since it has been reported that some CEACAM-binding respiratory bacteria upregulate the expression of their own receptor on host cells (24), the levels of individual CEACAM receptors endogenously expressed by infected A549 cells were assessed by Western blotting using anti‐CEACAM antibodies in time‐course experiments. No significant differences were observed in the expression of CEACAMs over time in infected A549 cells (data not shown).

Taken together, our results show that the binding of A. baumannii to CEACAM receptors on lung epithelial cells induces different CEACAM-specific cell signaling responses. A. baumannii cell invasion by CEACAM1 binding led to IL-8 secretion conceivably via NF-κB and Erk1/2-dependent pathways at 24 h postinfection. Conversely, A. baumannii invasion through CEACAM5 and CEACAM6 engagement was highly likely to proceed via LAP. Notably, at least a 1-log drop in the number of intracellular bacteria at 96 h postinfection was observed in all CEACAM-expressing cell lines, indicating that both pathways were effective in killing A. baumannii. Thus, its interaction with CEACAM1, CEACAM5, and CEACAM6 leads to more effective absorption and a longer intracellular stay, and subsequently, it is more effectively killed. Undoubtedly, further studies are needed in order to provide additional insights into the molecular mechanisms that lung epithelial cells use in response to A. baumannii infections.

## DISCUSSION

The present study identifies for the first time that human CEACAM1, CEACAM5, and CEACAM6 are exploited by A. baumannii for attachment to and invasion of lung epithelial cells. Typically, these members of the CEACAM family are present on the apical side of inner epithelial tissues throughout the human body, where they are involved in intercellular adhesion and in the regulation of several physiological and pathophysiological processes (21, 22, 42). Since more CEACAMs can be differentially expressed at the same time by human cells, adhesion to each CEACAM by A. baumannii was assessed by the use of human lung alveolar epithelial cells stably transfected with individual CEACAM receptors, a model system that has been used successfully before (20, 25, 29, 34). The CEACAM-binding ability reported here strongly suggests a convergent evolution of A. baumannii with other important pathogenic bacteria such as E. coli, N. gonorrhoeae, N. meningitidis, nontypeable H. influenzae, M. catarrhalis, *Salmonella* spp., H. pylori, and *Fusobacterium* spp. (20, 25, 43). This shared strategy confirms that the N domain of CEACAMs represents a particularly effective anchor for bacterial adhesion to human epithelia before being further exploited by pathogenic bacteria to be internalized in nonphagocytic cells via the zipper mechanism. Adhesion of bacteria to host cells could involve various protein-protein and protein-sugar interactions in a cooperative way. Instead, the internalization process implies the engagement of specific receptors, such as CEACAMs, by a specific bacterial ligand which induces receptor activation, thereby triggering actin cytoskeleton rearrangements to engulf and enclose bacterial pathogen into a membrane-bound vacuole (bacterial phagosome) (44, 45). A receptor-mediated internalization process for A. baumannii was previously reported; it was shown that A. baumannii binds the PAFR expressed on lung epithelial cells with the phosphorylcholine-containing porinD protein, thereby activating a cascade of pathways that eventually lead to bacterial entry into host cells (17). In addition, A. baumannii was shown to use a wide variety of molecules on host epithelial surfaces for adhesion and host colonization. A. baumannii Omps and autotransporters mediate adherence to components of the extracellular matrix (i.e., fibronectin, collagen, and laminin), as well as cell adhesion molecules, including integrins (46). It is noteworthy that H. influenzae and M. catarrhalis are also able to interact with PAFR, extracellular matrix, and cell adhesion molecules, in addition to CEACAMs (45). Therefore, like other pathogens, A. baumannii developed redundant mechanisms to adhere to and gain access into host cells that represent a nutrient-rich niche within which to replicate and promote further invasion and to avoid immune detection. It was reported that CEACAM-binding pathogenic bacteria are also able to induce a temporal upregulation of interacting receptors to promote their uptake into host cells (45). However, under our experimental conditions, no upregulation in the expression of CEACAMs was observed in A549-ev control cells following A. baumannii infection (data not shown).

Investigation of the initial steps of A. baumannii infection revealed that Rab5, Rab7, and LC3 were acquired by A. baumannii membrane-bound vacuoles. The small GTPases Rab5 and Rab7 play critical roles in phagocytosis being involved in the transport of pathogen-containing phagosomes to lysosomes for degradation (36, 47). The early endosome marker Rab5 is involved in the recruitment of the vacuolar protein sorting 34 (Vps34), class III phosphatidylinositol 3-kinase (PI3K-III), Rab7, and V-ATPase on its membranes (36). PI3K-III is a hetero-oligomeric complex that includes several accessory subunits, apart from Vps34, that generates phosphatidylinositol 3-phosphate (PI3P), a lipid second messenger that is recognized by several effector proteins responsible for membrane and cytoskeletal modifications (48). Instead, recruitment of Rab7 is required for endo-lysosomal traffic, while V-ATPase is required for progressive phagosomal acidification (36). Indeed, the use of a specific inhibitor of the V-ATPase, bafilomycin A1, led to a significant increase in the number of intracellular A. baumannii in all analyzed cell lines, indicating that, shortly after invasion, A. baumannii-containing vacuoles become more acidic due to V-ATPase activity. However, the number of intracellular A. baumannii in untreated cells increased from 24 to 48 h postinfection, while no increase could be seen from 48 to 72 h. Therefore, despite the action of V-ATPase and the poor tolerance of A. baumannii at low pH, the increase in the number of bacteria within the first 48 h after infection could be explained by the ability of A. baumannii to delay phagosomal acidification for at least 48 h regardless the presence or absence of CEACAMs, thereby preventing its killing. Several pathogens delay phagosomal fusion with lysosomes to achieve bacterial survival within host membrane-bound compartments (41, 49). It was shown that Burkholderia cenocepacia uses this survival mechanism to inhibit the V-ATPase, the main element responsible for phagosomal acidification, in macrophages (50). Moreover, B. cenocepacia cells survived in phagosomes for the first 4 h after infection, while they started to decline at 6 h postinfection before being killed by the phagosome-lysosome fusion (50). Although faster, this trend recalls our data on A. baumannii multiplication rates and survival within lung epithelial cells. Therefore, it can be speculated that such a delay could be helpful in nonprofessional phagocytic cells to A. baumannii to provide a way to escape and get access to deeper tissues, thereby promoting its spreading inside the host. In this scenario, another possible explanation could be that intracellular bacteria are sequestered in LC3-positive autophagic structures and can survive longer by triggering incomplete autophagy, as previously reported for A. baumannii (37).

**FIG 7:**
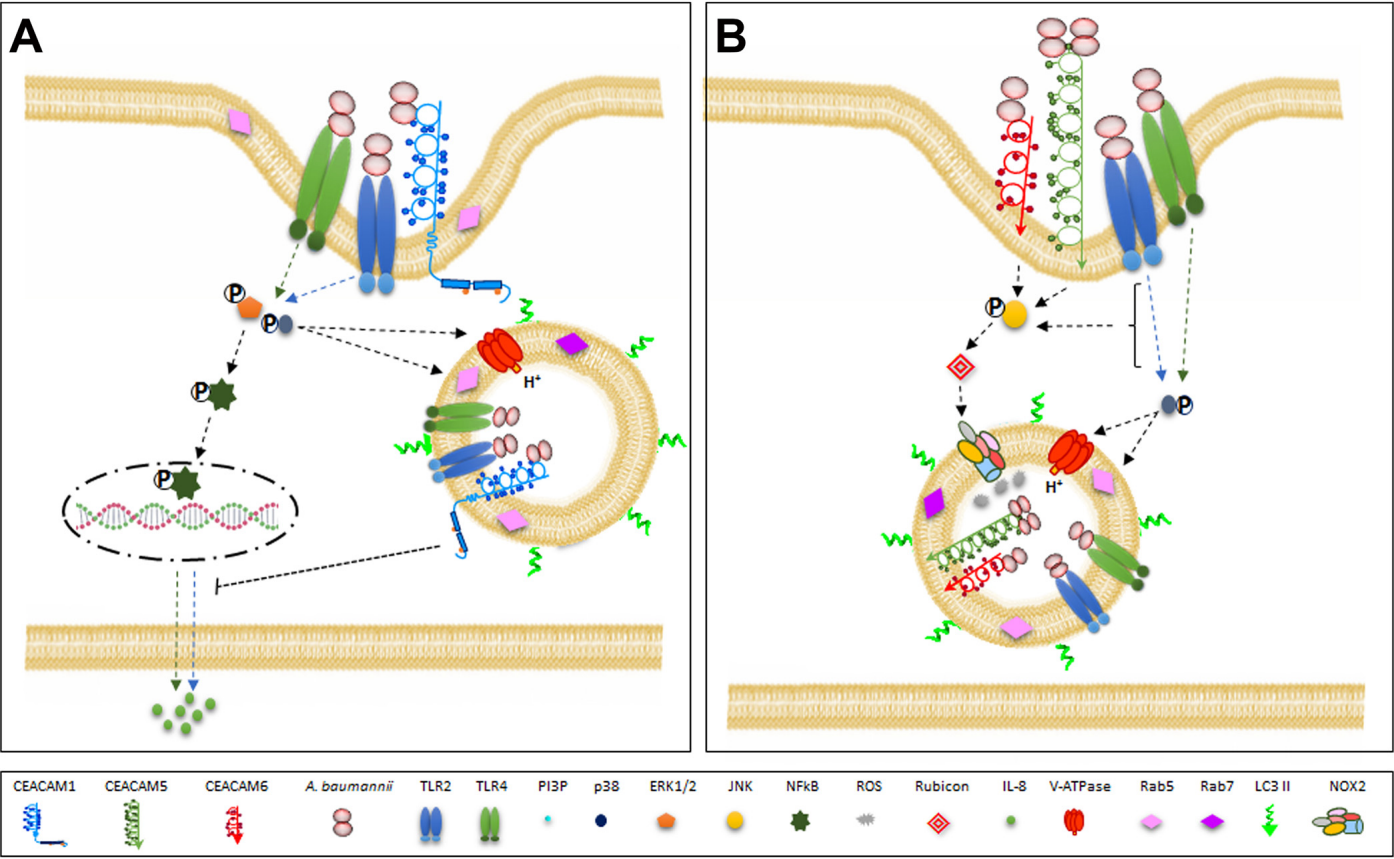
Working model of the signaling cascades triggered by the interaction of A. baumannii with CEACAM receptors expressed by transfected lung epithelial cells. The adhesion of A. baumannii cells to CEACAM receptors allows bacterial internalization within membrane-bound vacuoles that promptly become decorated by Rab5, Rab7, and LC3. Vacuoles become progressively acidic by recruitment of the V-ATPase, which pumps protons (H^+^) into the lumen using cytosolic ATP as an energy source. Moreover, phosphatidylinositol 3-kinase (PI3K) generates 3′-phosphoinositides, which accumulate in membrane-bound vacuoles to favor the acidification of vesicles. Activation of p38 enhances the retrieval of Rab5 via the activity of the guanine-nucleotide dissociation inhibitor (GDI), thereby increasing endocytic rates (67). (A) Proposed model for CEACAM1-mediated signaling process. Binding of A. baumannii to CEACAM1 as well as Toll-like receptors 2 (TLR2) and TLR4 triggers Erk1/2 phosphorylation, which in turn, activates NF-κB. Following nuclear translocation, NF-κB promotes transcription of the *IL-8* gene, thereby allowing a large amount of IL-8 secretion at 24 h postinfection (52). However, IL-8 secretion drops significantly in the A549 cell line expressing CEACAM1 at later time points, likely due to an inhibitory activity of its immune-receptor tyrosine-based inhibitory motif (ITIM) on the TLR2 signaling cascade. (B) Proposed model for the CEACAM5- and CEACAM6-mediated signaling process. Binding of A. baumannii to CEACAM5 or CEACAM6 as well as TLR2 and TLR4 triggers JNK1/2 phosphorylation, resulting in the recruitment of Rubicon, a component of the PI3K complex. Rubicon stabilizes the NOX2 complex, allowing it to produce reactive oxygen species (ROS) in the microbe-containing vacuole membranes (55). Both signaling pathways involving the intracellular trafficking of A. baumannii vesicles lead to acidification and lysosomal trafficking and, eventually, to microbial degradation. For simplicity, Rubicon is shown as a single protein. Individual components are not to scale.

Among other functions, CEACAM receptors were shown to regulate cellular signaling through homophilic or heterophilic interactions (20, 21, 23, 43). Therefore, by analyzing CEACAM signaling capacity on MAPKs, we found that CC-1 as well as A549-ev control cells displayed phosphorylation of Erk1/2 and NF-κB, which resulted in the secretion of IL-8. It is interesting to note that CC-1 secreted significantly more IL-8 at 24 h after infection than A549-ev control cells, while this secretion profile was completely reversed in the following time points. Previous studies outlined a significant increase of IL-8 upon A. baumannii lung infections in order to recruit and activate neutrophils for bacterial eradication, both *in vitro* and *in vivo* (51, 52). Based on our and previous data, it can be concluded that lipopolysaccharide (LPS) is recognized by Toll-like receptor 4 (TLR4), which activates NF-κB via Erk1/2, thereby leading to IL-8 secretion. Also, TLR2, which colocalizes with CEACAM1, was suggested to be involved in the same cell response (52, 53). Thus, the interaction between A. baumannii and the epithelial cells induced a strong production of IL-8 over the first 24 h. Therefore, although IL-8 secretion is initially induced by TLR2 and TLR4 signaling pathways, the dramatic reduction in IL-8 secretion at later time points observed in the CC-1 cell line could be mediated by the A. baumannii*-*CEACAM1 interaction; this interaction triggered a signaling pathway via the cytoplasmic ITIM domain of CEACAM1 that decreased cytokine release (Fig. 7). Interestingly, this immune-evasion mechanism was previously demonstrated for M. catarrhalis and N. meningitidis (29). In particular, it was demonstrated that engagement of CEACAM1 by UspA1 or Opa induced the phosphorylation of CEACAM1-L ITIM, resulting in the recruitment of the Src homology 2 domain-containing cytoplasmic protein tyrosine phosphatase 1 (SHP-1) to colocalized CEACAM1 and TLR2 (29). Consequently, the formation of the TLR2-PI3K class IA (PI3K I) complex induced by the pathogen is prevented, thereby inhibiting IL-8 secretion by inhibition of the PI3K I-Akt-NF-κB downstream signaling pathway (29). Further experiments are needed to define the molecular mechanisms involving TLR2 and TLR4 pathways as well as the mechanistic connection of NF-κB by which the A. baumannii-CEACAM1 interaction is able to upregulate IL-8 secretion. Conversely, a totally distinct signaling pathway was observed for CEACAM5 and CEACAM6 upon A. baumannii binding. Phosphorylated JNK1/2 as well as the expression of NOX2 and Rubicon were significantly increased in both CC-5 and CC-6 compared to CC-1 and A549-ev control cells. In the past years, it became clear that components of the autophagic machinery could be recruited to phagosomal membranes in an autophagy-related pathway named LC3-associated phagocytosis (LAP), in both phagocytes and epithelial cells (41, 54, 55). LAP pathways are characterized by LC3-decorated phagosomes and the presence of Rubicon and NOX2 complex, composed of p47phox, p40phox, p67phox, p22phox, and Rac1, the latter two considered hallmarks of LAP (54). This noncanonical form of autophagy is initiated by the engagement of a surface receptor such as TLR, Fc, or phosphatidylserine receptors (41, 54, 55). Activated receptors trigger the generation of PI3P through the PI3K complex, composed by VPS34, vacuolar protein sorting 15 (VPS15), Beclin-1, UVRAG, and Rubicon (41, 54, 55). PI3P is required for the recruitment of the ATG5-12 conjugation system and NOX2 stabilization (41, 54, 55). Rubicon stabilizes the p22phox and p40phox subunits for full activity of NOX2, which generates reactive oxygen species (ROS) for both bacterial killing and ATG5-12 activation to recruit LC3 to membrane phagosomes, possibly to enhance phagosome maturation (41, 54, 55). In line with other studies, our data show that LC3 levels increased early after infection, and it decorated A. baumannii membrane-bound vacuoles in all cell lines (37, 56). Moreover, only cell lines expressing CEACAM5 and CEACAM6 infected with A. baumannii displayed increased levels of phosphorylated JNK1/2 and Rubicon, leading us to hypothesize a JNK1/2-Rubicon cascade. Indeed, the only JNK1/2-Rubicon cascade was previously reported in nonalcoholic steatohepatitis (57). Therefore, we propose that cells expressing CEACAM5 and CEACAM6 induce LAP for clearance of A. baumannii via the JNK1/2-Rubicon-NOX2 pathway; expression of Rubicon guarantees the progression of LAP and inhibits, at the same time, the canonical autophagic pathway (41, 54, 55) (Fig. 7). On the other hand, when A. baumannii infects host cells in the absence of overexpressed CEACAM5 or CEACAM6, the activated TLR-CEACAM1 receptors trigger the Erk1/2-NF-κB signaling pathway, which in turn, leads to IL-8 secretion and possibly to the canonical autophagy, as reported previously (37, 53, 56). From 48 h after infection onward, LAP and autophagic pathways converge at the level of the lysosomes to kill the internalized bacteria. Interestingly, the occurrence of autophagy and LAP at the same time for infections caused by pathogens such as *Listeria* and *Salmonella* (55) was previously reported. Electron microscopic analyses of infected cells will definitively assess whether the CEACAM-dependent intracellular location of A. baumannii might have both single-membrane phagosomes and double-membrane autophagosomes.

It has been established that patients suffering from pulmonary diseases such as chronic obstructive pulmonary disease (COPD) have increased proinflammatory cytokines, which are known to upregulate the expression of CEACAM receptors on both epithelial and endothelial cells (24, 42, 58–60). Based on the data here, this condition can favor considerably the adhesion to and invasion of lung epithelial cells by A. baumannii. Indeed, CEACAM-binding pathogens such as H. influenzae and M. catarrhalis and, to a lesser extent, A. baumannii are the most commonly isolated bacteria from COPD patient specimens (61). Notably, nonseptic patients in ICUs also have high cytokine levels, as a consequence of a variety of tissue injuries, including trauma, surgery, or burns, as well as infections (62). Therefore, as shown for the effects of the upregulation of PAFR in smokers and COPD patients (63), we speculate that upregulation of CEACAMs could increase the risk of ventilator-associated or ICU-hospital-acquired pneumonia caused by A. baumannii in these patients. Further studies are needed to address this aspect of pathogenesis *in vivo*.

### Conclusions.

In the past years, A. baumannii has become a worrisome emerging pathogen, responsible for ventilator-associated pneumonia in ICUs. Unraveling the interactions between A. baumannii and host cells poses a great role in understanding its mechanism of pathogenesis and host risk factors leading to the infection. Thus, this study shows for the first time the interaction between A. baumannii and epithelial CEACAM receptors unraveling specific downstream signaling pathways that enable A. baumannii to enter alveolar host cell epithelia. Our results widen our knowledge of LAP, an autophagic-related pathway that epithelial cells use in response to pathogens interacting with CEACAMs. Moreover, our findings could have clinical implications in the management of patients suffering from pulmonary diseases as well as those in ICUs whose CAEACAM expression is upregulated. Further studies are needed to gain a better understanding of the roles of TLR2/TLR4 and CEACAMs as well as their cooperative activities, providing the basis for new therapeutic approaches to combat MDR A. baumannii infections.

## MATERIALS AND METHODS

### Bacterial strains and growth conditions.

The A. baumannii AB5075-UW strain used in this study was provided by BEI Resources (Manassas, VA). Routine growth and plating were carried out in Luria-Bertani broth (LB) and 1.5% agar plates (Difco, Milan, Italy). A single opaque colony distinguished under oblique lighting was inoculated in LB and grown at 37°C with vigorous shaking (200 rpm) to the mid-exponential phase (optical density at 600 nm [OD_600_] 0.8).

### Bacterial pulldown.

Approximately 2 × 10^8^ A. baumannii cells per ml were washed twice with phosphate-buffered saline (PBS) solution and incubated individually with 10 µg of recombinant human CEACAM1-Fc, CEACAM1ΔN-Fc, CEACAM5-Fc, CEACAM6-Fc, and CEACAM8-Fc (25) for 1 h at 37°C with head-over-head rotation. After incubation, bacteria were washed at least six times with PBS, pelleted, and either treated for immunofluorescent labeling or resuspended in cracking dye (2% SDS, 20% glycerol, 62.5 mM Tris-HCl, pH 6.8, 0.05% bromophenol blue, and 5% β-mercaptoethanol).

### Cell lines and transfection.

The human A549 lung epithelial cell type II line (ATCC CCL185 LGC Standards, Sesto San Giovanni, Italy) stably transfected with pdKCR-Neo vector (empty vector) or pdKCR-Neo-CEACAM1-L, pdKCR-Neo-CEACAM6, and pdKCR-Neo CEACAM8 (referred to as CC-1, CC-6, and CC-8, respectively) were used in this study (34). The A549-CEACAM5 was stably transfected with the pdKCR-dhfr-CEACAM5 vector (CC-5) (kindly provided by W. Zimmermann, Munich, Germany). The surface expression of each CEACAM in individual clones growing in the log phase was determined by flow cytometry (Fig. S1), as previously described utilizing the monoclonal antibody (MAb) 6G5j binding to CEACAM1, CEACAM3, CEACAM5, CEACAM6, and CEACAM8 (25, 64). In parallel, the absence of endogenous CEACAMs was analyzed by the monospecific anti-CEACAM1 (MAb B3-17), anti-CEACAM5 (MAb 3E10-3), anti-CEACAM6 (MAb 1H7-4B), and the anti-CEACAM8 (MAb 6/40c) staining. mRFP-GFP-LC3 HeLa cells stably expressing the chimeric LC3-GFP were a kind gift from D.C. Rubinsztein, Cambridge, UK. All cell lines were cultured in Dulbecco modified Eagle medium (DMEM) supplemented with 10% (vol/vol) heat-inactivated fetal bovine serum (FBS; Gibco, Milan, Italy), 2 mM l-glutamine, and 1 mg/ml Geneticin (G418, Sigma-Aldrich, Rome, Italy) at 37°C in a humidified atmosphere with 5% CO_2_.

### Bacterial adherence, invasion, and survival assays.

Semiconfluent cell monolayers of each cell line in the log phase were infected at a multiplicity of infection (MOI) of 1, centrifuged at 700 × *g* for 10 min, incubated for 2.5 h at 37°C in 5% CO_2_, and washed gently five times with PBS. A set of plates was lysed with 0.1% Triton X-100, and serially diluted lysates were plated on LB agar plates to determine the number of adherent bacteria (CFU/ml). The second and third sets of infected plates were used for fluorescent and immunofluorescent labeling. Following bacterial cell adhesion, the medium overlaying monolayers were replaced with fresh culture medium containing 3% FBS and 5 µg/ml of colistin sulfate (BioChemica, Milan, Italy) to kill extracellular bacteria, and cells were incubated further at 37°C in 5% CO_2_, as indicated. At each time point, cell monolayers were lysed, and the number of viable bacteria was determined by plating suitable dilutions on LB agar plates. Bacteria were also subcultured in the presence of colistin to rule out the onset of colistin-resistant bacteria as previously reported (65). Where indicated, bafilomycin A1 (Enzo Life Sciences, Milan, Italy) was added at the moment of infection (100 nM) and maintained for the adhesion time (2.5 h) and an additional 2 h in the presence of colistin sulfate.

### Fluorescent and immunofluorescent labeling.

Bacteria from the pulldown experiment were fixed with 4% paraformaldehyde with head-over-head rotation for 15 min, washed three times, and hybridized with 6G5j and secondary antibodies in PBS supplemented with 0.5% BSA (25). Finally, 200 µl of bacteria were centrifuged on polylysine-treated coverslips and mounted for microscopic analysis. Infected cell monolayers were fixed with 4% paraformaldehyde and stained with rhodamine-conjugated phalloidin and 4,6-diamidino-2-phenylindole (DAPI) following the manufacturer’s instructions (Cytoskeleton, Inc., and Molecular Probes, respectively, Milan, Italy) or immunostained. Briefly, cells were blocked with 1% bovine serum albumin (BSA) in PBS for 1 h at 37°C, washed with PBS, and incubated overnight at 4°C with 10 µg/ml of 6G5j antibody, as described above. After washing, cells were incubated with a TRITC-labeled goat anti-mouse IgG diluted 1:200 in PBS for 60 min at room temperature (RT) and washed three more times. Finally, samples were permeabilized for 10 min with a 0.25% solution of Triton X-100 in PBS, and bacterial and cellular DNAs were labeled with DAPI. For indirect immunofluorescence microscopy (LC3, Rab5, Rab7, and Rubicon), CC-1, CC-5, CC-6, and control cells were seeded on glass coverslips, infected at an MOI of 1, and incubated for the adhesion time (2.5 h) and for an additional 2 h in the presence of colistin sulfate. Cell monolayers were rinsed with PBS, fixed with methanol for 5 min at −20°C, and hydrated with PBS for 10 min at RT or, alternatively, fixed with 4% paraformaldehyde, permeabilized with 0.3% Triton X-100 for 5 min at RT, and blocked for 30 min in PBS-1% BSA at RT. Cell monolayers were incubated for 60 to 90 min at RT with rabbit anti-LC3 antibody (diluted at 1:400, Sigma), followed by incubation with either Alexa Fluor 647-conjugated anti-Rab5 or mouse anti-Rab7 (Santa Cruz, Milan, Italy), both diluted at 1:50 in PBS-1% BSA and Rubicon, diluted at 1:100 (Thermo Fisher Scientific, Milan, Italy). Anti-rabbit Alexa Fluor 488 (green) and anti-mouse Alexa Fluor 647 (red) were diluted at 1:400 and 1:250, respectively (Jackson Immunoresearch). LysoTracker Red DND-99 (Thermo Fisher Scientific) was used at 75 nM and incubated for 30 min at 37°C; subsequently, cell monolayers were fixed, permeabilized, and stained with DAPI. Images were acquired with a Leica DM5000B microscope equipped with the digital FireWire color camera system Leica DFX300 (Leica, Milan, Italy). Indirect immunofluorescence images of Rubicon were acquired using a Nikon Eclipse Ti2 confocal laser scanning microscope (Nikon, Florence, Italy). Images were digitally processed with Photoshop 6 (Adobe Systems Incorporated, CA, USA).

### Quantification of LC3 by in-cell Western assay.

Cells were seeded onto 24-well plates at semiconfluence and infected with strain AB5075 at an MOI of 1. At 1, 2.5, and 4.5 h postinfection (HPI), cells were gently rinsed with PBS and fixed with 50% methanol/acetone for 5 min at −20°C. The same protocol that was applied for LC3 indirect immunofluorescence was used, apart from the dilution factor of 1:2,000 of the rabbit anti-LC3 antibody (Sigma-Aldrich, Rome, Italy). Cell monolayers were incubated for 60 min at RT in the dark with the anti-rabbit-IRDye800 (green) and cellTag 700 (red) diluted in Odyssey blocking reagent 1:800 and 1:4,500, respectively. Cells were washed 3 times for 3 min in PBS, the excess liquid was removed, and plates were scanned using the Odyssey infrared imager (169 μm resolution, medium quality with 3 mm focus offset). Images and integrated fluorescence intensity, expressed as arbitrary units (a.u.), were acquired using LI-COR Image Studio 3.1 software.

### Gel electrophoresis and Western blot analysis.

Samples were denatured for 10 min at 95°C, and equal amounts of proteins (10 to 50 µg) were resolved either by 8% tricine-sodium dodecyl sulfate-polyacrylamide gel electrophoresis (T-SDS-PAGE) or 12% glycine SDS-PAGE and electrotransferred onto nitrocellulose membranes (Hybond-C, Millipore, Milan, Italy). Blots were probed with clone 6G5J, a monoclonal antibody that specifically recognizes human CEACAM1, 3, 5, 6, and 8, anti-CEACAM1 (MAb C5-1X, which binds to the A1B domain but not the N domain of human CEACAM1), anti-CEACAM5 (MAb 3E10-3), anti-CEACAM6 (MAb 1H7-4B), anti-CEACAM8 (MAb 6/40c) (25, 64), anti-phospho- and anti-ERK1/2 (Cell Signaling Technology), anti-phospho- and anti-p38 (Cell Signaling Technology and Santa Cruz, respectively), anti-phospho- and anti-JNK1/2, anti-phospho- and anti-p65, anti-NOX2, anti-GAPDH (all Santa Cruz), and anti-Rubicon (Thermo Fisher Scientific, Milan, Italy). Appropriate secondary antibody IgG conjugated to horseradish peroxidase was used (Bio-Rad, Milan, Italy). Blots were visualized with an enhanced chemiluminescence system (GE-Healthcare Bio-Sciences, Milan, Italy). The GAPDH was used as the loading control to check for equal protein loading in Western blot analysis (data not shown).

### Densitometric analysis.

Relative band intensities were quantified by densitometric analysis using ImageJ software (66). Quantification of the phosphorylation status is presented as the ratio of phosphorylated/total protein, while NOX2 and Rubicon were normalized to GAPDH. Results are expressed as arbitrary units.

### Interleukin-8 (IL-8) release.

Cell supernatants from different A549 CEACAM transfected cell lines infected with strain AB5075 for 24, 48, and 72 h were analyzed with a sandwich enzyme-linked immunosorbent assay (ELISA; Thermo Fisher Scientific, Milan, Italy) to determine IL-8 concentrations.

### Statistical analyses.

Normal distribution was determined with the Shapiro-Wilk test. The statistical differences of normally distributed data were analyzed with one-way analysis of variance (ANOVA) and *post hoc* Student’s *t* test. Values of *P < *0.05 were taken as being statistically significant.
