# Supplementary figures and images for: Acinetobacter baumannii Targets Human Carcinoembryonic Antigen-Related Cell Adhesion Molecules (CEACAMs) for Invasion of Pneumocytes
Source: mSystems. 2020 Dec 22;5(6):e00604-20. doi: 10.1128/mSystems.00604-20 (PMC7762790; doi:10.1128/mSystems.00604-20)

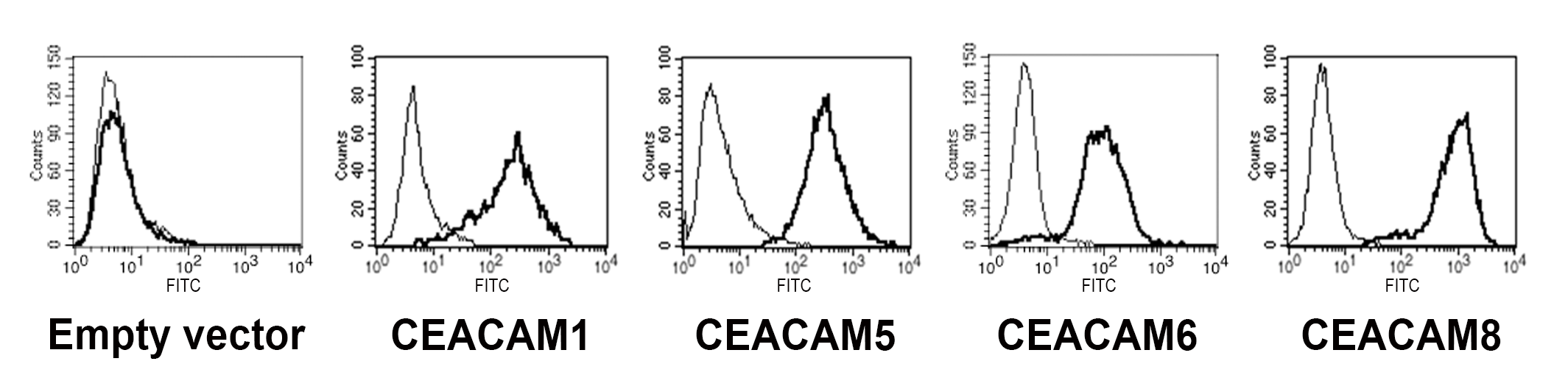

Supplement: FIG S1 [file mSystems.00604-20-sf001.tif]

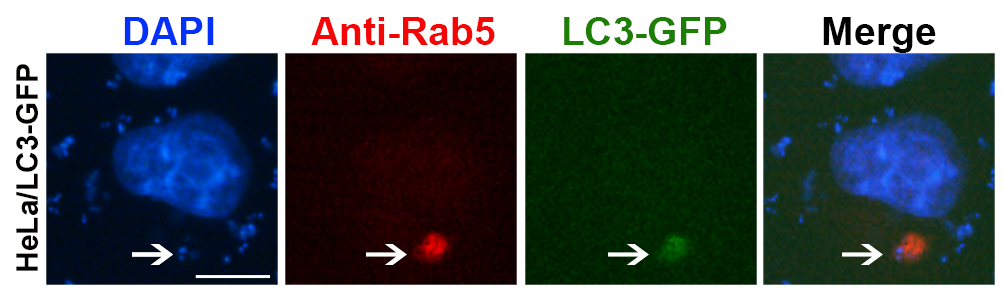

Supplement: FIG S2 [file mSystems.00604-20-sf002.tif]

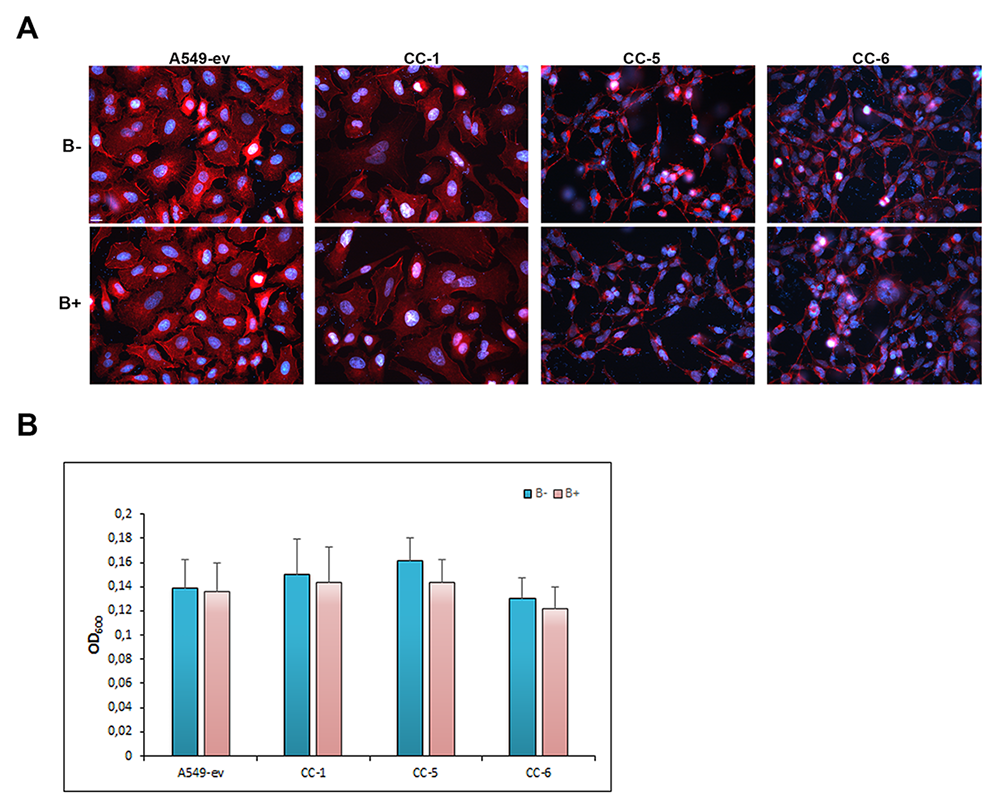

Supplement: FIG S3 [file mSystems.00604-20-sf003.tif]
